# Fibroblasts from different sites may promote or inhibit recruitment of flowing lymphocytes by endothelial cells

**DOI:** 10.1002/eji.200838232

**Published:** 2009-01

**Authors:** Helen M McGettrick, Emily Smith, Andrew Filer, Stephen Kissane, Michael Salmon, Christopher D Buckley, G Ed Rainger, Gerard B Nash

**Affiliations:** 1 Centre for Cardiovascular Sciences, The Medical School, The University of BirminghamBirmingham, UK; 2 Centre for Immune Regulation, The Medical School, The University of BirminghamBirmingham, UK

**Keywords:** Adhesion, Endothelial cells, Fibroblasts, Lymphocytes

## Abstract

We examined the hypothesis that stromal fibroblasts modulate the ability of endothelial cells (EC) to recruit lymphocytes in a site-specific manner. PBL were perfused over HUVEC that had been cultured with fibroblasts isolated from the inflamed synovium or the skin of patients with rheumatoid arthritis or osteoarthritis, or from normal synovium, with or without exposure to the inflammatory cytokines TNF-α+IFN-γ. Fibroblasts from inflamed synovium, but no others, caused unstimulated HUVEC to bind flowing lymphocytes. This adhesion was supported by α_4_β_1_-VCAM-1 interaction and stabilised by activation of PBL through CXCR4–CXCL12. Antibody neutralisation of IL-6 during co-culture effectively abolished the ability of EC to bind lymphocytes. Cytokine-stimulated EC supported high levels of lymphocyte adhesion, through the presentation of VCAM-1, E-selectin and chemokine(s) acting through CXCR3. Interestingly, co-culture with dermal fibroblasts caused a marked reduction in cytokine-induced adhesion, while synovial fibroblasts had variable effects depending on their source. In the dermal co-cultures, neutralisation of IL-6 or TGF-β caused partial recovery of cytokine-induced lymphocyte adhesion; this was complete when both were neutralised. Exogenous IL-6 was also found to inhibit response to TNF-α+IFN-γ. Normal stromal fibroblasts appear to regulate the cytokine-sensitivity of vascular endothelium, while fibroblasts associated with chronic inflammation bypass this and develop a directly inflammatory phenotype. Actions of IL-6 might be pro-inflammatory or anti-inflammatory, depending on the local milieu.

## Introduction

Vascular endothelial cells (EC) regulate leukocyte recruitment during inflammation. In response to cytokines such as TNF-α or IFN-γ, EC up-regulate expression of selectins and members of the immunoglobulin superfamily and generate and present chemokines, thus promoting leukocyte adhesion and migration 1, 2. The local physiochemical microenvironment, including haemodynamic forces and sub-endothelial stroma, can influence these endothelial responses, thus modulating leukocyte recruitment 3, 4. Of particular interest here is the ability of stromal cells to induce or modify the pro-inflammatory responses of EC.

*In vivo*, EC are rarely found in isolation but are usually adjacent to pericytes and other stromal cells including fibroblasts and smooth muscle cells (SMC). Recent studies by ourselves and others show that stromal cells can strongly influence endothelial recruitment of several types of leukocyte 5–9. Culturing human HUVEC and bronchial epithelial cells together on opposite sides of a filter promoted neutrophil transmigration towards IL-8 6, while dermal fibroblasts from scleroderma patients promoted transmigration of a T-cell line through an immortalised HUVEC line 5. Using flow-based models, we found that co-culturing HUVEC with secretory SMC augmented the capture of neutrophils, monocytes and lymphocytes induced by TNF-α 7. In addition, crosstalk between hepatic sinusoidal EC and hepatocytes induced lymphocyte adhesion in the absence of exogenous cytokines and amplified recruitment in response to lymphotoxin 8. Most recently, we demonstrated that HUVEC cultured with fibroblasts from the synovium of patients with rheumatoid arthritis (RA) supported the capture of flowing neutrophils in the absence of exogenous cytokines, a response not seen with dermal fibroblasts 9.

There is growing evidence that fibroblasts are a heterogeneous population that demonstrate topographic and positional memory, even within a single tissue 10–12. This led us to propose that there might be a ‘stromal post-code’ for fibroblasts from different anatomical sites, which reflected their potential to differentially influence leukocyte recruitment and subsequent fate in inflammation 4. Here, we set out to evaluate this concept by testing whether fibroblasts could directly induce lymphocyte recruitment by EC, or modulate the responses of EC to cytokines, depending on the origin of the fibroblasts. Our studies indicated that ‘normal’ fibroblasts from non-inflamed tissue could modulate the ability of cytokine-treated HUVEC to recruit flowing lymphocytes, while fibroblasts associated with chronic inflammation (from the arthritic synovium) could directly induce lymphocyte adhesion. Interestingly, we detected differential effects of IL-6 generated in these co-cultures on lymphocyte recruitment, depending on the co-culture and cytokine milieu.

## Results

### Adhesion of flowing lymphocytes to endothelial-fibroblast co-cultures

**Figure 1:**
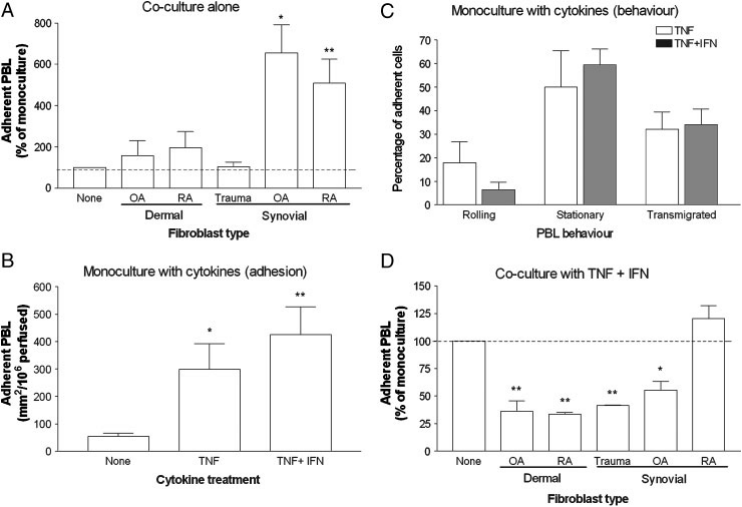
Lymphocyte adhesion to EC cultured alone or with fibroblasts, with or without cytokine treatment. (A) Adhesion to unstimulated EC cultured alone (None) or co-cultured with dermal or synovial fibroblasts from patients with RA or OA, or with synovial fibroblasts from a patient suffering from mechanical trauma. (B) Adhesion to endothelial mono-cultures unstimulated or stimulated with TNF-α or TNF-α+IFN-γ. (C) Behaviour of lymphocytes adherent to endothelial mono-cultures stimulated TNF-α or TNF-α+IFN-γ (analysed 10 min after washout). (D) Endothelial mono- or co-cultures stimulated with TNF-α+IFN-γ. Data are mean±SEM from 3 to 11 independent experiments, using at least 3 different donors for each cell type, except for ‘trauma’ where fibroblasts from a single donor were tested on 2 occasions. In all experiments, co-cultures were compared with mono-cultures (None), and data are expressed as a percentage of that observed in the paired control (None), which itself was normalised to 100%. ^*^p<0.05, ^**^p<0.01 for comparison to ‘None’ by paired *t-*test.

EC and fibroblasts were co-cultured on opposite sides of transwell filters, and the filters were cut out and incorporated into a parallel plate flow chamber allowing the direct visualisation of lymphocyte adhesion to EC using phase contrast microscopy. Initially we examined whether fibroblasts could induce endothelial responses in the absence of exogenous cytokines. Unstimulated EC mono-cultures supported only low levels of lymphocyte adhesion from flow (Fig. 1A). Interestingly, synovial fibroblasts from inflamed joints of patients with RA or osteoarthritis (OA) induced lymphocyte adhesion to co-cultured EC in the absence of exogenous cytokines (Fig. 1A). The great majority of these adherent lymphocytes (∼90%) were stationary, firmly adherent cells, and a small sub-population were rolling (2.2±0.8 or 12.8±9.3% of adherent cells were rolling on EC co-cultured with RA or OA synovial fibroblasts, respectively; mean±SEM for *n*=13 or 3 experiments). A sub-population of the adherent cells had migrated through the endothelial monolayer by 10 min after washout (7.6±2.0 or 18.5±9.3% of adherent cells migrated through EC co-cultured with RA or OA synovial fibroblasts respectively; mean±SEM for *n*=13 or 3 experiments). In contrast, lymphocyte adhesion to EC cultured with dermal fibroblasts from the same donors was not significantly above that observed on mono-cultures, and synovial fibroblasts from a patient undergoing knee surgery due to mechanical injury (trauma) also failed to up-regulate adhesion to EC (Fig. 1A).

Next, we examined whether the crosstalk between fibroblasts and EC during co-culture could alter the endothelial responses to cytokines. On the basis of previous work by ourselves and others (13–15; see also *Materials and methods*), we initially studied adhesion induced by 100 U/mL of TNF-α alone, or combined with 10 ng/mL of IFN-γ. Lymphocytes adhered efficiently to EC stimulated with either treatment (Fig. 1B). While a significant proportion of adherent cells rolled on the surface (∼20%), most were stationary and firmly adherent, and a significant proportion (∼30%) went on to migrate through the endothelial monolayer (Fig. 1C). In agreement with previous studies 13, 15, the cytokine combination tended to support greater adhesion and more firm adhesion, and so was used in further co-culture experiments. In this model, the ‘non-stimulatory’ fibroblasts from the skin of patients with RA or OA, or from normal (trauma) synovium, all markedly reduced the number of lymphocytes adhering to cytokine-stimulated EC compared with stimulated mono-cultures (Fig. 1C). Synovial fibroblasts from RA patients tended to increase the endothelial responses (although not significantly), but synovial fibroblasts from OA patients inhibited lymphocyte adhesion in the cytokine-treated co-cultures, although to a slightly lesser extent than the dermal fibroblasts (Fig. 1C). The lymphocytes that did bind to the cytokine-treated EC co-cultured with dermal fibroblasts were nevertheless firmly adherent, with <10% rolling.

The above data suggest that although fibroblasts from chronically inflamed synovium induce inflammatory responses, healthy ‘resting’ fibroblasts (dermal or synovial) limit the endothelial response to cytokines. In all further experiments we compared the genetically matched dermal and synovial fibroblasts isolated from RA patients.

### Effect of blocking adhesion or chemokine receptors on lymphocyte adhesion

**Figure 2:**
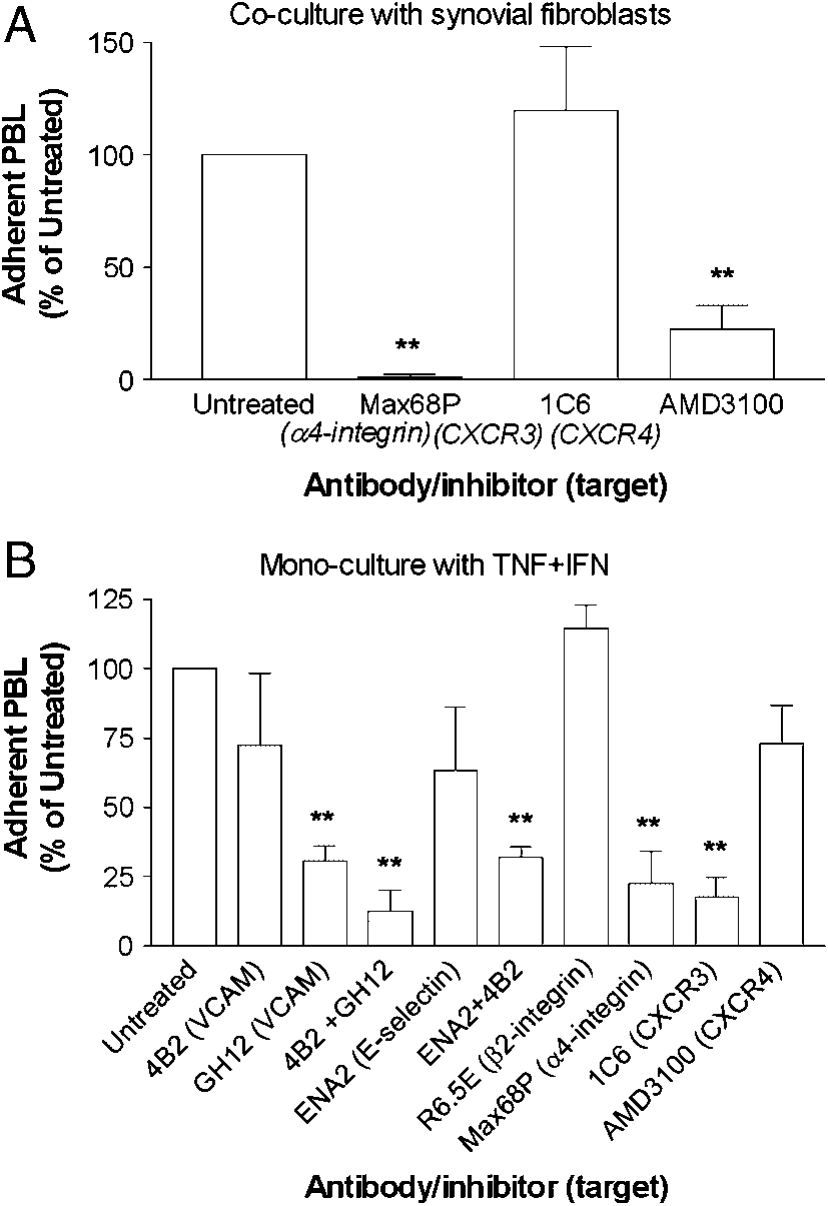
Effects of blocking adhesion receptors and chemokines on lymphocyte adhesion to (A) unstimulated endothelial-RA synovial fibroblast co-cultures or (B) endothelial mono-cultures treated with TNF-α+IFN-γ. HUVEC were treated with antibodies against E-selectin (ENA-2) and/or VCAM-1 (4B2 and GH12, against domains 1 and 4, respectively) for the last 30 min of the 24 h cytokine treatment. Lymphocytes were incubated for 15 min with antibodies against α_4_-integrin (Max68P), CXCR3 (1C6), β_2_-integrins (R6.5E) or with the CXCR4 inhibitor, AMD3100, before perfusion. Alternatively, Lymphocyte adhesion was expressed as a percentage of control (untreated), which itself was normalised to 100%. Data are mean±SEM from at least three independent experiments. Not all antibodies were used in all experiments, but treated cells were always compared with paired untreated controls. ^**^p<0.01 for comparison to untreated by paired *t*-test.

We examined the mechanisms supporting adhesion of lymphocytes to unstimulated EC cultured with synovial fibroblasts. Lymphocytes were treated with function-blocking antibodies against α_4_-integrin or CXCR3 (which binds IFN-γ-inducible chemokines), or with an antagonist of CXCR4 (receptor for CXCL12/stromal derived factor-1α (SDF-1α)) before perfusion. Adhesion was essentially abolished when α_4_-integrins were blocked and greatly reduced when CXCR4 was inhibited, but blockade of CXCR3 was ineffective (Fig. 2A).

Subsequently, we analysed lymphocyte adhesion to cytokine-treated endothelial mono-cultures after treatment with function-blocking antibodies against key adhesion and chemokine receptors. Pre-treatment of EC with antibody against domain 1 of VCAM-1 caused slight but non-significant reduction, antibody against domain 4 had a greater and significant effect, while blockade of both domains reduced adhesion by 88% (Fig. 2B). Antibody against E-selectin also tended to reduce adhesion. Although this effect was not significant, when treatment was combined with antibody against domain 1 of VCAM-1, there was a significant reduction in adhesion. When lymphocytes were treated with antibody against β_2_-integrin there was no reduction in adhesion, but blockade of α_4_-integrin reduced adhesion markedly, to a level close to that when both domains of VCAM-1 were blocked (Fig. 2B). Thus, the interaction between α_4_-integrin and VCAM-1 was the main mechanism for binding (capture and firm adhesion) of flowing lymphocytes, although E-selectin might also have contributed in the capture phase. In contrast to the studies with unstimulated co-cultures, anti-CXCR3 antibody treatment caused a significant (∼80%) reduction in lymphocyte adhesion to cytokine-stimulated EC, while inhibiting CXCR4 had no significant effect (Fig. 2B).

Collectively, these data suggest that initial lymphocyte binding and firm adhesion were supported by interaction of α_4_β_1_-integrin with VCAM-1 in both models. However, lymphocyte adhesion was stabilised by activation through CXCL12 in the unstimulated system, while CXCR3 ligands were responsible for stabilisation in the cytokine-treated model.

### Effect of co-culture on mRNA and surface expression of E-selectin and VCAM-1 by EC

**Figure 3:**
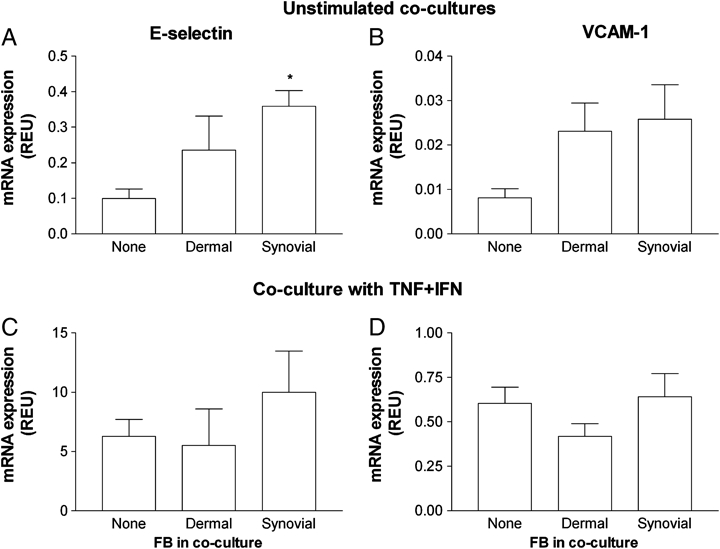
Effect of RA co-culture on the mRNA expression of E-selectin or VCAM-1 by EC. Endothelial mRNA was isolated from (A and B) unstimulated or (C and D) cytokine (TNF-α+IFN-γ) treated co-cultures and gene expression of (A and C) E-selectin or (B and D) VCAM-1 was assessed by qPCR. In (A) and (B), ANOVA shows a significant effect of co-culture on E-selectin expression (*p*<0.05) and borderline significance of co-culture on VCAM-1 expression (*p*=0.08). Values were expressed as REU compared with β-actin. Data are mean±SEM from three independent experiments, using three different donors for each cell type. ^*^p<0.05 compared with None by Dunnett's test.

To further investigate the mechanisms by which fibroblasts modulated lymphocyte adhesion to endothelium, we used quantitative real-time PCR (qPCR) and flow cytometry to examine the effect of fibroblasts on the mRNA and surface-protein expression of E-selectin and VCAM-1 by co-cultured EC. Low levels of mRNA for E-selectin and VCAM-1were detected in unstimulated EC, but expression was dramatically up-regulated following cytokine treatment (Fig. 3). Co-culture caused a slight up-regulation of E-selectin and VCAM-1 mRNA expression by unstimulated EC, but this effect was similar for each type of fibroblast from RA donors, and expression remained far below levels for cytokine-treated cells (Fig. 3A and B). Co-culture with either type of fibroblast had no consistent effect on mRNA expression of E-selectin or VCAM-1 by cytokine-stimulated EC (Fig. 3C and D).

**Figure 4:**
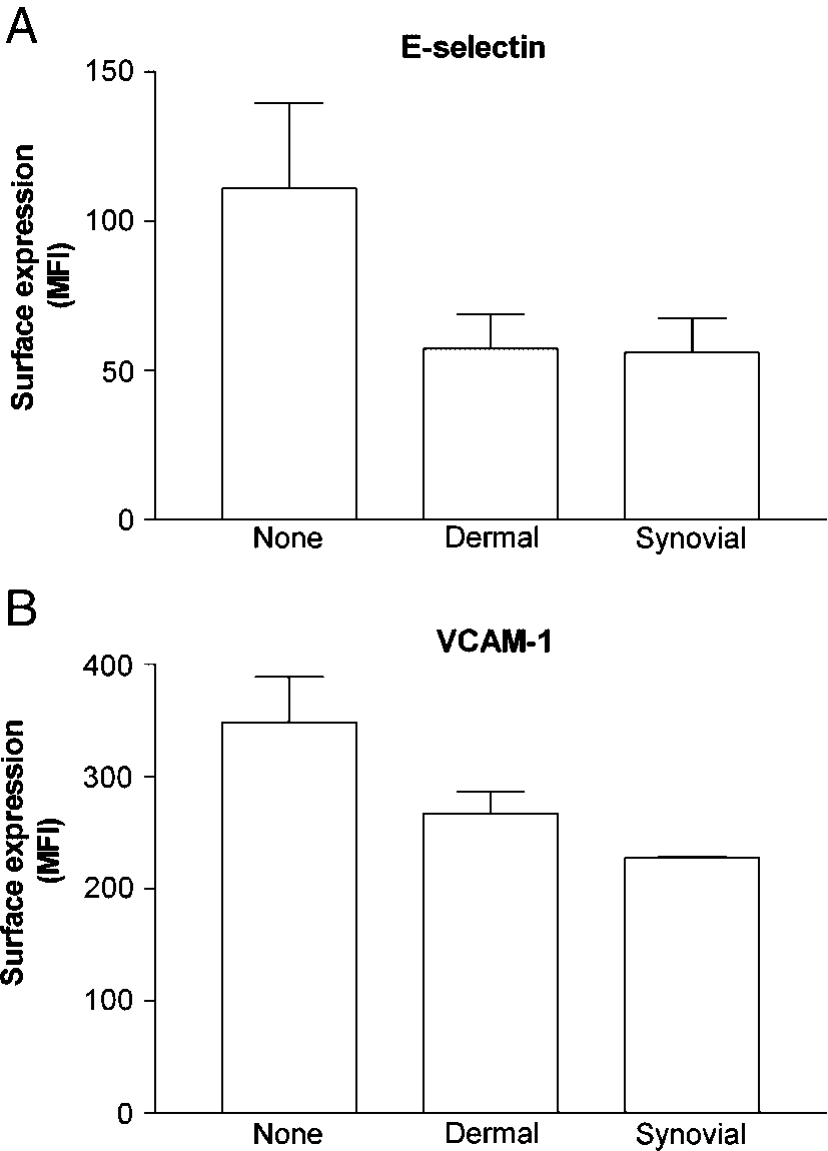
Effect of RA co-culture on the surface expression of E-selectin or VCAM-1 by EC. EC from cytokine treated co-cultures were stained with antibodies against (A) E-selectin or (B) VCAM-1 and surface expression was assessed by flow cytometry (expressed as MFI). Data are mean±SEM from three independent experiments, using three different donors for each cell type.

Judged by flow cytometry, surface levels of E-selectin and VCAM-1 were barely above background for unstimulated EC co-cultured with either fibroblast type (data not shown). Cytokine-treated EC showed strong expression of these receptors, and there was a tendency towards a reduction in E-selectin and VCAM-1 surface expression on co-cultures compared with EC cultured alone (Fig. 4). However, these effects were not statistically significant and similar trends were observed for each type of fibroblast from RA donors.

Overall, changes in mRNA and surface expression of the identified endothelial capture receptors did not show clear differences between effects of co-culture with dermal or synovial fibroblasts, or correlate with functional changes.

### Effect of co-culture on mRNA expression of chemokines by EC

Data presented above demonstrated a major role for CXCL12 (SDF-1) in the increased adhesion of lymphocytes to unstimulated EC co-cultured with synovial fibroblasts. Previously we showed that synovial fibroblasts expressed significantly more CXCL12 mRNA than dermal fibroblasts 16. Here, we evaluated CXCL12 mRNA levels in EC using qPCR. Unstimulated and cytokine-stimulated EC expressed very low levels of mRNA for CXCL12 (relative expression units (REU) <0.001 relative to β-actin in each case). Although co-culture with either type of fibroblast from RA donors significantly increased these levels in the absence of cytokines (REU=0.006±0.001 or 0.003±0.001 for dermal or synovial co-cultures, respectively; mean±SEM *n*=3, *p*<0.05 compared with EC alone by Dunnett's test), the levels remained low and were not significantly different between the fibroblasts. In the presence of cytokines, the levels of CXCL12 mRNA were not significantly affected by culture with fibroblasts (data not shown).

CXCR3 ligands were important in lymphocyte adhesion to cytokine-treated EC. Thus we analysed the effect of co-culture on the expression of the ligands for CXCR3 by EC using RT-PCR. High levels of mRNA for the CXCR3 ligands, CXCL9 (Mig), CXCL10 (IP-10) and CXCL11 (I-TAC) were detectable in EC treated with TNF-α+IFN-γ, but these levels were not modified by co-culture with either fibroblast type (data not shown).

Collectively, the above data showed no effects of co-culture on the expression of chemokines by EC that correlated with the recruitment patterns we observed with either unstimulated or cytokine-treated cells.

### Requirement for direct contact between EC and fibroblasts

Next, we analysed whether direct contact between EC and fibroblasts from RA patients was required to generate the different lymphocyte adhesion patterns. Fibroblasts were cultured in the bottom of the plate, while EC were grown inside the filters, thereby ensuring no direct contact. Unstimulated EC that had been cultured in the presence of synovial fibroblasts bound lymphocytes efficiently from flow (103±32 cells/mm^2^/10^6^ perfused *versus* 30±9 cells/mm^2^/10^6^ perfused for EC mono-cultures; mean±SEM; *n*=3). Dermal fibroblasts from RA patients again inhibited the response to TNF-α+IFN-γ treatment, with adherent lymphocyte being reduced by 52±10% (mean±SEM; *n*=3) compared with cytokine-treated endothelial mono-cultures (*p*<0.05 by paired *t*-test). The presence of synovial fibroblasts without contact did not significantly alter adhesion of lymphocytes to the cytokine-treated EC (data not shown). Collectively, these findings suggest that both the intrinsic pro-inflammatory effects of co-culture with synovial fibroblasts and the ability of dermal fibroblasts to suppress inflammatory responses arose from the release of soluble mediators. The magnitude of the responses induced without contact (noted above) tended to be less than that shown in Figure 1 where cells were only separated by a porous filter. Thus, close juxtaposition of fibroblasts with EC may potentiate the activity of such mediators, but direct contact did not appear to be essential.

### Roles of IL-6 or TGF-β in modulation of lymphocyte adhesion

**Figure 5:**
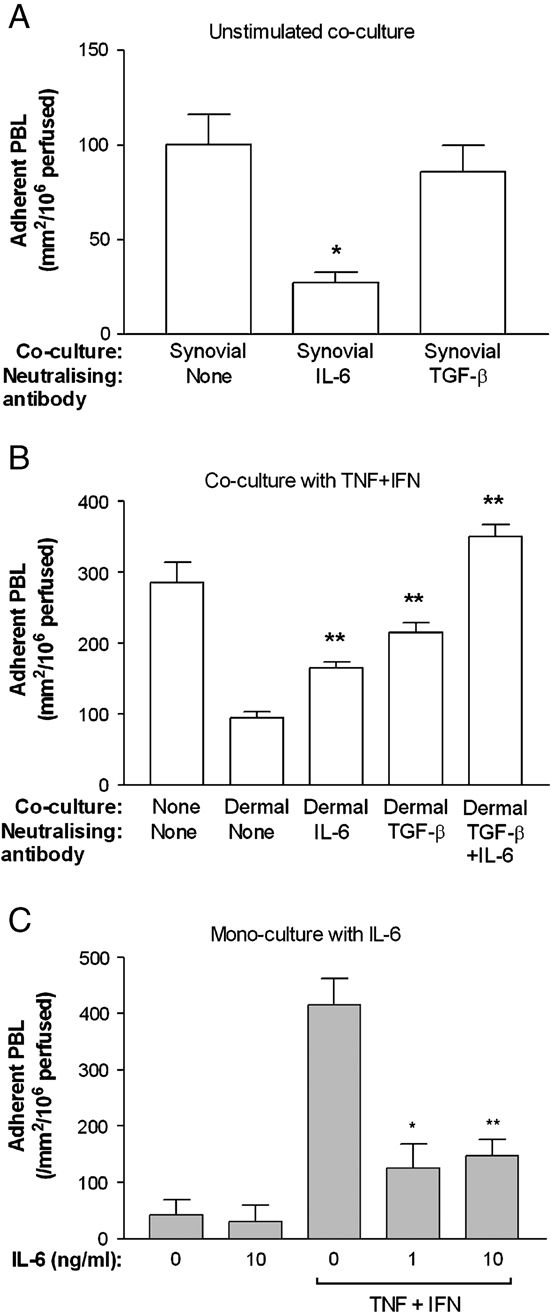
Effect of neutralising IL-6 or TGF-β, or of adding IL-6 on lymphocyte adhesion. (A) Neutralisation in unstimulated endothelial-RA synovial fibroblasts co-cultures; (B) neutralisation in cytokine-stimulated endothelial-RA dermal fibroblasts co-cultures; (C) addition of IL-6 to unstimulated or cytokine-stimulated EC mono-cultures. Data are mean±SEM from at least three experiments, using at least three different donors for each cell type. In all cases, ANOVA showed a significant effect of treatment on lymphocyte adhesion (*p*<0.01). ^*^p<0.05 and ^**^p<0.01 by Bonferroni test compared with co-cultures without neutralisation in (A) and (B), or to cytokine-treated mono-culture without IL-6 in (C).

We previously identified IL-6 as a soluble promoter of the adhesion of neutrophils in a similar unstimulated co-culture model 9. Here, significantly more IL-6 was produced in endothelial co-cultures with synovial fibroblasts than in co-cultures with dermal fibroblasts from the same RA donors in the absence of exogenous cytokines (2.92±0.52 ng/mL compared with 0.64±0.14 ng/mL, respectively; mean±SEM *n*=9, *p*<0.01 by paired *t*-test). Neutralising antibodies against IL-6 or TGF-β were added to the co-cultures for 24 h before the assay. Neutralisation of IL-6 caused a significant reduction in lymphocyte adhesion to EC cultured with synovial fibroblasts, while neutralisation of TGF-β had no significant effect (Fig. 5A).

Next we investigated the agent(s) suppressing responses to cytokines in co-cultures of RA dermal fibroblasts with EC. In this case, neutralisation of IL-6 or TGF-β in co-cultures caused a partial recovery of lymphocyte adhesion towards the level in cytokine-treated mono-cultures, and recovery was complete when both IL-6 and TGF-β were neutralised (Fig. 5B). Neutralising IL-6 or TGF-β had no effect on lymphocyte adhesion to cytokine-stimulated EC cultured with synovial fibroblasts (data not shown).

Since soluble IL-6 appeared to play a role in inducing adhesion in unstimulated co-cultures, and in inhibiting adhesion in cytokine-treated co-cultures, we tested the effects of adding IL-6 to EC. When exogenous IL-6 (10 ng/mL) was added to otherwise unstimulated endothelial mono-cultures, it failed to induce EC to support lymphocyte adhesion (Fig. 5C). On the other hand, treating EC for 24 h with IL-6 (at 1 or 10 ng/nL; concentrations similar to those detected in either dermal or synovial co-cultures) prior to the addition of TNF-α+IFN-γ dramatically reduced lymphocyte adhesion in response to the cytokines (Fig. 5C).

In general, IL-6 signals in cells through ligation of a compound receptor comprised Gp130 (CD130) and IL-6Rα (IL-6 receptor alpha; CD126) (17, 18, for reviews). Gp130 is a component of several dimeric receptors for different cytokines and is expressed in many cells including EC. However, IL6Rα has more restricted expression and others have indicated that IL-6 has no detectable effect on the responses of HUVEC unless soluble sIL-6Rα is present 19. We checked the expression of Gp130 and IL-6Rα mRNA in EC using qPCR, with or without co-culture with fibroblasts. The expression of Gp130 was about 100 times higher than IL-6Rα in EC (REU relative to 18 S was 1.6±0.2×10^−4^ *versus*. 1.4±10^−6^, respectively; mean±SEM from six measurements; two each from EC cultured alone, with dermal or with synovial fibroblasts, with no consistent differences between mono-cultures and co-cultures). Nevertheless, IL-6Rα was always detected by qPCR before cycle 35 in the six measurements, suggesting a low level of mRNA was present. We thus tested whether IL-6Rα was present on EC using immunofluorescence and flow cytometry. In two experiments, surface IL-6Rα was not detectable for mono-cultures or co-cultures (MFI relative to IgG1 control=0.92±0.05; mean±SEM from six measurements; two each from EC cultured alone, with dermal or with synovial fibroblasts,) and in two experiments MFI relative to IgG1 control was just detectable for mono-cultures or co-cultures (MFI relative to IgG1 control=1.42±0.07). Thus, IL-6Rα mRNA and surface protein were near the limits of detectability in the EC in mono-culture or co-culture.

### Microarray analysis of gene transcription by EC during co-culture

**Figure 6:**
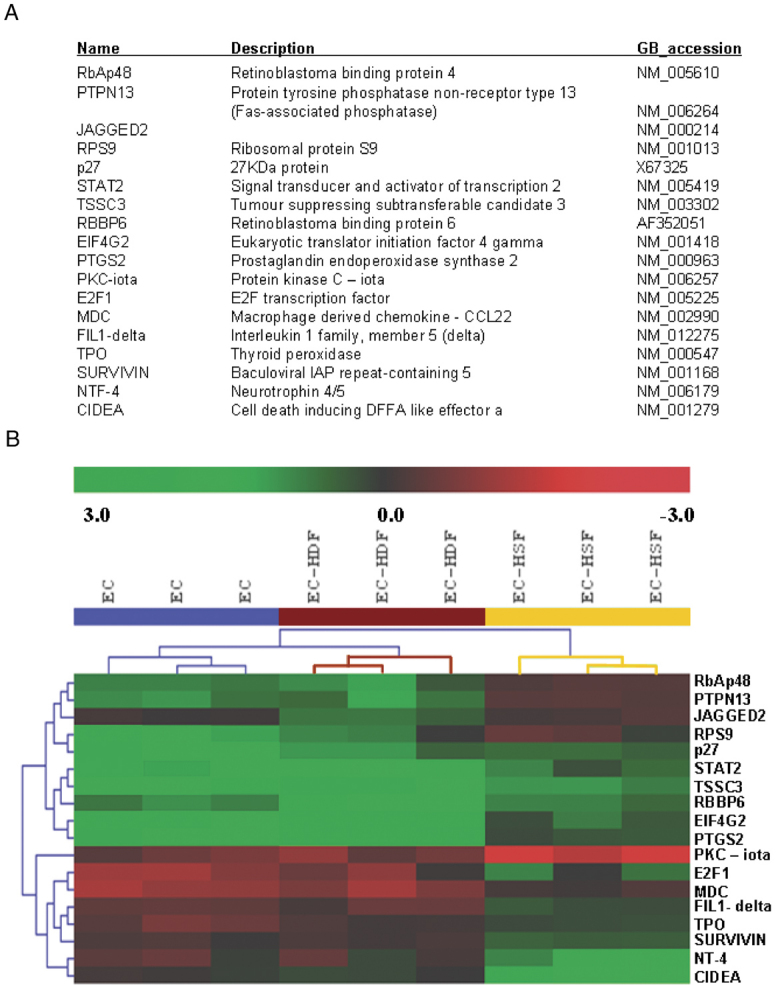
Effect of co-culture on the gene transcriptional profiles of EC. EC were cultured alone or with human RA dermal or synovial fibroblasts (HDF or HSF respectively) for 24 h in the absence of exogenous cytokines. Endothelial mRNA was isolated and analysed using microarray technology on an 850 human gene array. Data was normalised (sample/reference) and then analysed by TIGR Multiple Experiment Viewer allowing statistical analysis of microarrays (SAM) and hierarchical cluster analysis (HCA). The SAM false-positive level was set at 0%. (A) List of 18 genes identified by SAM to vary significantly between the three culture-conditions. (B) HCA based on the 18 genes identified. The colour scale from green to red for each gene represents the natural logarithm of the gene expression, relative to the value for the reference sample tested with it. The different culture conditions showed distinct segmentations, as indicated by the coloured tree (blue-brown-yellow) across the top, associated with them. Comparisons between the different culture conditions were done on three occasions, each using different primary EC isolates and different arrays. In each array, the individual genes were tested in triplicate.

The functional responses induced or modified by co-culture were not clearly linked to changes in the expression of single genes (either for adhesion molecules, chemokines or IL-6 receptors). Nevertheless, IL-6 was implicated in distinct and diverse effects for the different co-cultures, suggesting that the underlying state of the EC may have been altered under the different culture conditions. To investigate this possibility, we used DNA microarray technology to examine changes in the transcriptional profiles of EC cultured alone or with the different fibroblasts from RA donors. The microarray analysed 850 immune-response genes (associated with adhesion and chemokine receptors, cytokines, apoptosis, cell cycle and intracellular signalling pathways). Our aim was to test whether the cells had taken on separate genomic ‘inflammatory’ phenotypes, rather than to identify specific genes that might be linked to altered function. Indeed, at a 0% false-positive level, ‘statistical analysis of microarrays’ (SAM) indicated that there were 18 genes that varied significantly between the three culture-conditions (Fig. 6A). Subsequent hierarchical clustering analysis (HCA) based on these genes showed distinct segmentation between the transcriptional profiles of the endothelial mono- and co-cultures (Fig. 6B). Interestingly, EC co-cultured with synovial fibroblasts displayed a transcriptional phenotype further removed from EC cultured alone than EC co-cultured with dermal fibroblasts (Fig. 6). This trend was borne out by individual comparisons of culture conditions, where 79 genes showed significant differences between EC cultured alone or with synovial fibroblasts, and only two genes were significantly different between EC cultured alone or with dermal fibroblast (data not shown). Thus, microarray analysis indicated that EC cultured with RA synovial fibroblasts had developed a separate phenotype from EC cultured with dermal fibroblast and from those in mono-culture.

## Discussion

Fibroblasts, once believed to be relatively inert cellular components of the tissue, are now believed to play an important role in tissue homeostasis, in particular, in regulating leukocyte recruitment in inflammation 4, 10. Here we have demonstrated for the first time that fibroblasts can stimulate recruitment of lymphocytes, or limit this response in EC, depending on the tissue from which the fibroblasts were obtained. When fibroblasts from the synovium of patients with RA or OA were cultured with EC, adhesion of flowing lymphocytes was induced even in the absence of added cytokines. In contrast, dermal fibroblasts from the same donors, and fibroblasts from non-inflamed synovium, had little effect on adhesion to otherwise unstimulated EC, but actually reduced the number of lymphocytes binding in the presence of cytokines TNF-α+IFN-γ. In the cytokine-stimulated model, the fibroblasts from inflamed synovium were less consistent in their effects, with those from RA patients tending to be stimulatory, but those from OA patients being inhibitory. The responses induced by synovial or dermal fibroblasts from RA patients appeared to be largely caused by soluble mediators. These may have been made more effective by close juxtaposition of cells, but direct contact between cells was not essential. Interestingly, IL-6 had opposing effects depending on the milieu: it was essential for the stimulatory effects of synovial fibroblasts in the absence of added cytokines, but in the presence of TNF-α+IFN-γ, IL-6 along with TGF-β mediated the inhibitory effects of dermal fibroblasts.

Adhesion of lymphocytes to EC cultured with RA synovial fibroblasts was essentially abolished by blockage of α_4_-integrin and greatly reduced by inhibition of CXCR4, the receptor for CXCL12 (SDF-1). This is consistent with previous studies showing that interaction between α_4_β_1_-integrin and VCAM-1 can support capture of flowing lymphocytes by EC and that activation of this integrin is required to stabilise adhesion 20, 21. Although CXCL12 has been shown to fulfil such an integrin-activating and adhesion stabilising role when added exogenously 22, this is the first report of activity of endogenous, cell-generated CXCL12. We previously found that CXCL12 was constitutively expressed and secreted by rheumatoid synovial fibroblasts 16. Since CXCL12 mRNA was barely detectable in EC here, and there are no reports of endogenous CXCL12 activity in HUVEC, it is highly likely that CXCL12 was transferred from the synovial fibroblasts and presented by the EC during co-culture. We found only slight up-regulation of VCAM-1 in co-cultures, while CXCR4 ligation was required for most of the adhesion seen in synovial co-cultures. This suggests that the receptors used for initial capture from flow were only slightly increased and that activation of lymphocyte integrins by CXCL12 from synovial fibroblasts was the critical factor for the elevation of stable adhesion. An analogous situation occurred in our previous studies, where synovial fibroblasts induced selectin-dependent adhesion of neutrophils to EC 9. There, selectin expression was again barely detectable and stable adhesion required activation of CXCR2 on the neutrophils.

When EC were stimulated with TNF-α+IFN-γ, lymphocyte adhesion was greatly reduced by combined blockade of domains 1 and 4 of VCAM-1. Blockade of α_4_-integrin on lymphocytes caused a similar degree of inhibition of adhesion. Thus for co-culture, it is likely that the interaction between α_4_β_1_-integrin and VCAM-1 was the main basis of lymphocyte adhesion. Fibronectin has been shown previously to contribute to adhesion of lymphoid cells lines to HUVEC, but not PBL 23. We attempted to quantify the level of fibronectin on the surface of our cultures by immunofluorescence labelling and flow cytometry, but did not detect a signal above that obtained with non-specific control antibody (data not shown). Thus, given the efficacy of the combined VCAM-1 blockade, no other ligand for PBL integrin need be postulated here. The finding that blockade of domain 4 of VCAM-1 was more effective than domain 1 was unexpected based on some previous reports. Domain 1 has been suggested to act as the main capture receptor from flow 24, 25, but domain 4 may be important in adhesion strengthening for activated lymphocytes 26. The data presented here mainly represent firmly adherent lymphocytes that have remained attached for minutes, and so our data suggest that domain 4 may be important for maintaining adhesion. E-selectin has also been shown to make a contribution to adhesion of PBL to TNF-α-treated HUVEC 27 and there was evidence of a minor contribution to adhesion here. Combination of antibodies against E-selectin and domain 1 of VCAM-1 had an effect greater than either antibody alone, suggesting both assisted in capture.

In the cytokine model, activation through CXCR3 was important to stabilise adhesion, and EC showed strong mRNA expression of three potential ligands for CXCR3 (CXCL9, 10 and 11). These results are consistent with previous reports demonstrating that CXCR3 ligands promote lymphocyte adhesion to TNF-α+IFN-γ-stimulated HUVEC 13. Cultured synovial fibroblasts have been shown to secrete CXCL9, CXCL10 and CXCL11 following stimulation with IFN-γ alone 28 or in combination with TNF-α *in vitro* 29 and this might contribute to the augmentation of adhesion in synovial co-cultures. However, down-regulation of adhesion by co-culture with dermal fibroblasts was not associated with reduction in the expression of these chemokines. Although surface expression of VCAM-1 or E-selectin showed a tendency to be reduced, this was evident in the co-cultures with synovial fibroblasts as well. Thus while inhibition of the response to cytokines in the dermal co-cultures might arise from changes in secretion or presentation of chemokines, no firm conclusion can be made.

Soluble mediators, in particular IL-6, played important roles in the modulatory effects of fibroblasts. Rheumatoid synovial fibroblasts are known to secrete a range of pro-inflammatory cytokines, including IL-6 30. Here, RA synovial co-cultures consistently secreted higher concentrations of IL-6 than dermal co-cultures in the absence of cytokines. Neutralisation of IL-6 significantly inhibited lymphocyte adhesion to EC cultured with synovial fibroblasts, as was the case in earlier studies of neutrophils adhesion to similar co-cultures 9. However, when we added exogenous IL-6 to EC alone, this did not induce an increase in lymphocyte adhesion. This is consistent with work by Modur *et al*. 19, who found that addition of soluble IL-6Rα in addition to IL-6 was required to induce adhesion of neutrophils to EC. Previously, we did not detect sIL-6R in co-culture supernatants 9. In fact, although the co-receptor Gp130 is widely expressed among different cells, IL-6Rα is believed to be restricted in expression to mononuclear leukocytes, fibroblasts and hepatocytes 31, 32. Nevertheless, we examined IL-6Rα mRNA expression by qPCR and found detectable levels, albeit much lower than those for Gp130. Neither was modified by co-culture with synovial or dermal fibroblasts. This led us to evaluate the surface expression of IL-6Rα protein on EC by flow cytometry, where very low levels were detected in two cultures but labelling was not above background in two others. Again, this was the same for mono-cultures or co-cultures. The foregoing indicates that it is unlikely that the IL-6 acting alone directly induced increased adhesion to EC in the synovial co-cultures. IL-6 may have induced the synovial fibroblasts to increase the production of CXCL12 and/or other agents promoting lymphocyte recruitment. However, another possibility is that EC in the modified phenotype induced by synovial co-culture were more responsive to IL-6 than simple mono-cultures even though there was no evident change in the expression of receptors (see below).

In contrast, IL-6 took on an inhibitory role in conjunction with TGF-β, in the reduced response to cytokines induced by co-culture of EC with dermal fibroblasts. Interestingly, we found that addition of exogenous IL-6 greatly reduced the response of endothelial mono-cultures to TNF-α+IFN-γ. Such an inhibitory effect of IL-6 has not been reported in EC previously to our knowledge and suggests that the low levels of IL-6Rα, which we detected, could modify cellular responses at least. TGF-β has been reported to suppress the secretion of chemokines, including CXCL10, from HUVEC stimulated with TNF-α+IFN-γ 33. In addition, pre-incubating human dermal microvascular EC with TGF-β inhibited their response to TNF-α or IL-1, and abrogated mononuclear leukocyte adhesion 34. *In vivo*, intra-tracheal injection of IL-6 with TGF-β inhibited neutrophil extravasation into LPS-induced lung inflammation 35, indicating that the combination can act as an inhibitor of acute inflammation. Here, IL-6 may have modulated responses of EC to cytokines and may also have induced dermal fibroblasts to generate inhibitory accessory signals, including TGF-β, which reduced the ability of EC to bind lymphocytes (*e.g*. by altering chemokine trafficking and/or presentation as noted above). Synovial fibroblasts released at least as much IL-6 as dermal fibroblasts, but the endothelial response to TNF-α+IFN-γ was not decreased in their presence. Thus it appears either that the co-cultured EC were in an altered phenotype that had a different response to IL-6 (as suggested above) or that secondary inhibitory agents were not released by the synovial cells. In any case, the results indicate that a single agent such as IL-6 can have quite different effects on an inflammatory response depending on the local environment and presence of other growth factors or cytokines.

Because the effects of IL-6 were disparate in the different types of co-culture, and changes in leukocyte recruitment were not simply attributable to changes in the expression of adhesion molecules or chemokines by the EC, we wondered whether the EC took on distinctive phenotypes when cultured with synovial or dermal fibroblasts. To investigate this we carried out gene microarray analysis for endothelial mono-cultures and co-cultures. The analysis indicated that although the differently cultured EC were distinguishable by their gene expression profiles, the synovial fibroblasts modulated a much greater number of genes than the dermal fibroblasts, and they induced a genomic state that was much more distinct from the EC cultured alone. This suggests that the fibroblasts from inflamed tissue not only provided CXCL12 and IL-6 but induced a phenotype in the EC, which was responsive to IL-6 and adhesive to lymphocytes. On the other hand, dermal fibroblasts left EC in a state that was not intrinsically responsive to IL-6. Instead, we hypothesise that the dermal fibroblasts provided IL-6, which moderated the response to cytokine treatment (an effect reproduced in mono-cultures), rather than establishing a new, non-responsive phenotype in advance.

In conclusion, these studies reveal that stromal cells can modulate the inflammatory response mediated by EC and that this modulation may itself depend on the state of the stromal cells in question (*e.g*. their own exposure to a chronically-inflamed environment). Not only fibroblasts but hepatocytes and SMC can also modulate the ability of overlying EC to recruit leukocytes 7–9. The effects may be directly stimulatory, or modulatory of responses to other agonists. It appears that fibroblasts from non-inflamed stroma can regulate the cytokine-sensitivity of vascular endothelium, while fibroblasts associated with chronic inflammation bypass this and develop a directly inflammatory phenotype. In the context of RA, the effects of synovial fibroblasts in co-culture are consistent with reports that CXCL12 is expressed highly in the synovium of the rheumatoid joint and may promote T-cell recruitment 36–38. IL-6 has been implicated as a modulator of RA in humans 39 and in animal models 40, while in rats, inhibition of α_4_-integrin reduced the accumulation of T cells into arthritic joints 41. Thus the model described here appears relevant to the pathology of RA in particular. More generally, co-cultures of appropriate primary cells, with or without added cytokines, offer a means to study physiological processes regulating inflammation in human tissue. The results in such systems may be complex as illustrated by the effects of IL-6 here, which depended on the local milieu and other released factors. It is thus difficult to predict *a priori* which soluble mediators should be added, *e.g*. to model inflammation using endothelial mono-cultures, and further studies are needed to fully define the regulatory or stimulatory repertoires of stromal cells.

## Materials and methods

### Isolation of human PBL, fibroblasts and EC

Venous blood from healthy individuals was collected in EDTA tubes (Sarstedt, Leicester, UK) and PBL were isolated using histopaque 1077 followed by panning on culture plastic to remove contaminating monocytes 42. PBL were washed, counted, and adjusted to a final concentration of 2×10^6^/mL in PBS containing Ca^2+^ and Mg^2+^ (Gibco Invitrogen Compounds, Paisley, Scotland), 0.15% BSA and 5 mM glucose (Sigma, Poole, UK) (PBSAG).

Tissue samples from synovium and overlying skin were obtained from the same patients with RA or with OA, and from the synovium of a patient undergoing surgery for mechanical injury (trauma), and fibroblasts were isolated as described previously 43. Cells were cultured in RPMI 1640 (Gibco) supplemented with 10% heat inactivated FCS, MEM-non-essential amino acids, 1 mM sodium pyruvate, 2 mM l-glutamine, 100 U/mL penicillin and 100 μg/mL streptomycin (all from Sigma) and used between passages 5 and 9.

HUVEC were isolated from umbilical cords as described previously 44 and cultured in M199 supplemented with 20% FCS, 10 ng/mL epithelial growth factor, 35 μg/mL gentamycin, 1 μg/mL hydrocortisone (all from Sigma) and 2.5 μg/mL amphotericin B (Gibco). HUVEC were used because they were the only primary human cells available in sufficient quantities at early passage, for the large number of studies described here. This was especially the case as we used first passage cells without expansion, which might change responsiveness. In addition, we and others have characterised the ability of HUVEC to bind leukocytes and to respond to cytokines in great detail. Although studies with microvascular EC would be interesting, HUVEC may be considered as a widely used ‘generic’ EC capable of responding to a wide range of stimuli. All human tissues were obtained with informed consent and with approval from the local research ethics committee.

### Culture of EC alone or with fibroblasts on filter inserts

Fibroblasts and EC cultures were dissociated using trypsin/EDTA (Sigma) and co-cultured on the opposite sides of 6-well, low-density, 0.4 μm pore Transwell filter inserts (BD Pharmingen, Cowley, UK). Fibroblasts (7×10^5^) were seeded onto inverted filters and cultured for 24 h. HUVEC were then seeded on the inner surface of inserts at a concentration that yielded confluent monolayers within 24 h. For comparison, parallel cultures of HUVEC alone were seeded inside inserts. The cells were cultured in fibroblast medium for 48 h. When desired, 100 U/mL of TNF (Sigma) was added, alone or with 10 ng/mL of IFN-γ (Peprotech, London, UK), for the last 24 h. The cytokine concentrations and combination were based on published studies in which IFN-γ potentiated T-cell recruitment induced by TNF-α 13, 15, our own titrations of TNF-α in adhesion studies (*e.g*. 14) and preliminary studies, which verified that combination of TNF-α and IFN-γ was more effective for inducing PBL adhesion then either alone. In some experiments, EC were cultured alone for 24 h and then treated with 1 or 10 ng/mL of IL-6 (Immunotools, Friesoythe, Germany) for 24 h prior to cytokine-stimulation as above, where the IL-6 was present during the cytokine-stimulation.

In studies to investigate the requirement for direct contact between co-cultured cells, fibroblasts were seeded onto the bottom of a 6-well plate for 24 h, after which HUVEC were seeded inside the filters. The cells were then cultured in the same plate for 24 h prior to cytokine-stimulation as above.

### Incorporation of filters into a parallel plate flow chamber

Filters were incorporated into a parallel plate flow chamber and connected to a perfusion system mounted on the stage of a phase-contrast videomicroscope held at 37°C, as described previously 45. PBL were perfused over the HUVEC for 4 min, followed by washout with cell-free PBSAG, all at a wall shear stress of 0.1 Pa. After 2 min of washout, video recordings lasting ∼20 s were made of a series of fields of known area along the central axis of the flow chamber. In each field, lymphocytes bound to the EC were counted, and each was classified as either (i) rolling adherent (spherical cells moving over the surface much slower than free-flowing cells); (ii) stationary or firmly adherent (typically with distorted shape and actually migrating slowly on the surface); or (iii) transmigrated under the endothelial monolayer (phase dark and spread). In some cases, video recordings were also made after 10 min washout, and lymphocyte behaviour re-evaluated. The total number of adherent lymphocytes (*i.e*. all three behaviours) was averaged *per* field and expressed/mm^2^/10^6^ cells perfused 46.

### Antibody treatments

In some experiments, HUVEC were treated for 20 min with the following: ENA2 (anti-E-selectin F(ab) fragment, 1 μg/mL; BD); 4B2 (anti-VCAM-1, recognising immunoglobulin domain 1, 10 μg/mL; R&D Systems, Abingdon, UK) or GH12 (anti-VCAM-1, recognising domain 4, 10 μg/mL; kind gift from Dr. Roy Lobb) alone or in combination. It may be noted that VCAM-1 has two potential sites for ligation of α_4_β_1_-integrin, and both may need to be blocked to ablate lymphocyte interaction with EC depending on the stimulatory regime 24, 47. Alternatively, neutralising antibodies against IL-6 (clone 6708, 5 μg/mL; R&D) or TGF-β (clone 1825, 5 μg/mL; R&D) were added when co-culture was established. In other experiments, lymphocytes were treated for 15 min with 10 μg/mL of the following: 1C6 (anti-CXCR3; R D); Max68P (anti-α_4_-integrin) or R6.5E (anti-β_2_-integrin) (both gifts from Dr. Tony Shock, Cell Tech, Slough, UK). The above are IgG1 antibodies previously shown to block functions 7, 9, 15, 24, 47–49. Alternatively, lymphocytes were treated for 15 min with 1 mg/mL of the CXCR4 inhibitor, AMD3100 (AnorMED, British Columbia, Canada).

### Flow cytometry of endothelial surface receptors

EC on filters were incubated with non-conjugated antibodies against E-selectin (1.2B6) or VCAM-1 (1.4C3; both Dako, Ely, UK) or with PE-conjugated antibody against IL-6Rα/CD126 (M91; Immunotools, UK) for 30 min at 4°C. Mouse IgG1 or PE-conjugated mouse IgG1 (both Dako) were used as the negative controls. For non-conjugated primary antibodies, samples were washed for 5 min with ice-cold PBS containing 4% BSA prior to incubation with goat anti-mouse FITC-conjugated secondary antibody (Dako) for 30 min at 4°C. All samples were washed and incubated with enzyme-free cell dissociation buffer (Sigma) for 30 min. Cells were retrieved, washed and analysed using a Coulter XL flow cytometer. Data were expressed as MFI.

### mRNA quantification by PCR

Trypsin/EDTA was used to detach HUVEC from the inside of filters, and mRNA was isolated from the cells using the RNeasy Mini Kit (Qiagen, Crawley, UK). mRNA levels of CXCL9, CXCL10, and CXCL11 chemokine were analysed by RT PCR. Primers were synthesised by Alta Biosciences (University of Birmingham, UK) as described previously 15. Amplified products were run on 1.25% agarose gel containing ethidium bromide, analysed by densitometry and data expressed as percentage of β-actin band density.

E-selectin, VCAM-1, ICAM-1, CXCL12, CD126, CD130, β-actin and 18 S mRNA were analysed by qPCR using Quanti-Tect™ probe RT-PCR kit according to manufacturer's instructions (Qiagen). ICAM-1, VCAM-1, E-selectin and CD126 FAM-labelled primers, and β-actin and 18 S VIC-labelled primers were bought as Assay on Demand kits from Applied Biosystems (Warrington, UK). CD130 FAM-labelled primers were from Eurogenetec (Southampton, UK). CXCL12 (SDF-1) FAM labelled forward (5′-GCC-CGT-CAG-CCT-GAG-CTA), reverse (3′ GAC-GTT-GGC-TCT-GGC-AAC-A) and probe sequences (AGA-TGC-CCA-TGC-CGA-TTC-TTC-GAA-A) were synthesised by Alta Biosciences. Samples were amplified using the 7900 HT Real-Time PCR machine and analysed using the software package SDS 2.2 (Applied Biosystems). Data were expressed as REU relative to β-actin (for E-selectin, VCAM-1, ICAM-1 and CXCL12) or 18 S (for CD126 and CD130).

### mRNA analysis by custom microarray

Custom DNA microarrays analysing 850 immune-response genes (associated with adhesion and chemokine receptors, cytokines, apoptosis, cell cycle and intracellular signalling pathways) were synthesized as described previously 50. Total mRNA was extracted from detached HUVEC using an RNAqueous kit according to manufacturer's instructions (Ambion, Huntington, UK). Extracted RNA was reverse transcribed and cDNA was amplified using the SMART cDNA synthesis kit following the manufacturer's protocol (BD Biosciences). The cDNA was purified using QIAquick PCR purification kit following manufacturer's instructions (Qiagen), using 30 μL of water for the final elution, and stored at −20°C. In-house reference sample was constructed by mixing cDNA from human buffy coat, fibroblasts, EBV-infected cells and peripheral blood mononuclear cells. An aliquot of 500 ng of cDNA from sample and reference was labelled by direct incorporation of dCTP, conjugated with Cy3 and Cy5, respectively. Microarrays were hybridized overnight at 42°C (Corning, USA), washed and scanned with GenePix 4000 microarray scanner (Axon Instruments, CA, USA). Each gene was analysed in triplicate in each experiment. Data were normalised (sample/reference) using the Gene Expression Pattern Analysis Suite v3.1 software package, and then exported to TIGR Multiple Experiment Viewer (http://www.tigr.org/software), allowing SAM and HCA. In analyses reported here, the SAM false-positive level was set at 0%.

### Statistical analysis

Variation between multiple treatments was evaluated using ANOVA, followed by Dunnett's test (for comparison to control) or Bonferroni multiple comparison post-test as appropriate. Differences between individual treatments were evaluated by paired *t*-test. *p*<0.05 were considered statistically significant.

## Abbreviations

EC: endothelial cells
HCA: hierarchical clustering analysis
OA: osteoarthritis
qPCR: quantitative real-time PCR
RA: rheumatoid arthritis
REU: relative expression units
SAM: statistical analysis of microarrays
SDF-1α: stromal derived factor-1α
SMC: smooth muscle cells
